# Immunosuppression by hemorrhagic fever-causing viruses

**DOI:** 10.18632/oncotarget.6509

**Published:** 2015-12-08

**Authors:** Bjoern Meyer, Hinh Ly

**Affiliations:** Department of Veterinary and Biomedical Sciences, University of Minnesota, Twin Cities, MN, USA

**Keywords:** viral hemorrhagic fevers, arenaviruses, ebola, dengue, immunosuppression

The outcomes of human virus infections are tightly linked to cellular immune responses, which are initiated early in the course of the infection by the activation of the innate immune pathways. The typical recognition of viral infections by the innate immune system occurs *via* the cytoplasmic recognition of viral nucleic acids by cellular receptors, such as the RIG-I-like receptors (RLRs) RIG-I and MDA5. Following the initial detection, these RLRs activate the type I interferon (IFN) induction pathway resulting in the activation of transcription factors, i.e. interferon regulatory factor 3 (IRF3), IRF7 or nuclear factor kappa-light-chain-enhancer of activated B cells (NFκB). This results in the expression and secretion of type I IFNs (i.e., IFNα and INFβ), which bind to their cellular receptors on the membrane in an autocrine and paracrine manner to initiate the IFN-signaling pathway. Downstream of this pathway, additional transcription factors, acting on the IFN-stimulated response element (ISRE), are activated, resulting in the expression of hundreds of IFN-stimulated genes (ISGs) to ultimately set up the antiviral stage [1].

Some members of four families of viruses (*Arenaviridae, Filoviridae, Flaviviridae*, and *Bunyaviridae*) can cause deadly hemorrhagic fever (HF) infections in humans. Lassa virus (LASV) of the *Arenaviridae* family, for example, accounts for an estimated half a million HF infections annually [2], second only to the number of annual Dengue infections worldwide [3]. There are currently no FDA-licensed therapeutics or vaccines available for many of these HF-causing viruses. HF viral replication and disease progression that can lead to severe symptoms, such as vascular leakage, hemorrhages and multi-organ failure, usually originate with a strong inhibition of IFN-induction and/or -signaling by these viruses [4].

It appears that immune suppression by these viruses is not perfect as early IFN-responses are often triggered. However, the dose dependent suppression of type I IFNs by viral IFN-antagonists expression eventually leads to an effective inhibition of the innate immune system resulting in high viral load towards late stages of the infection. The HF-causing viruses utilize multiple of their proteins to inhibit IFN-induction and/or IFN-signaling pathways in order to increase the efficacy of immunosuppression.

The IFN-induction pathway resembles an amplification cascade that shifts the attention for viral IFN-antagonists towards the reduction and inhibition of the early stages of this pathway by reducing the pathogens associated molecular pattern (PAMP) RNAs as well as reducing the activation of the RLRs. We have recently shown that LASV and other pathogenic arenaviruses encode two IFN-antagonists, namely NP and Z proteins, on their relatively small genomes [5, 6]. NP is a 3′-5′ exoribonuclease that is capable of destroying PAMP RNAs and therefore blunt the recognition of infection by the RLRs. It has also been shown to directly bind to the cellular IKKε protein to prevent IRF3 phosphorylation. At the same time the Z protein of pathogenic arenaviruses binds to the CARD domains of RIG-I and MDA5 to prevent their downstream interactions with the mitochondrial antiviral signaling protein (MAVS) and therefore inhibits the IFN-induction pathway. Inhibition of this pathway seems paramount for HF viruses as all of these viruses target the induction of type I IFNs to eventually prevent proper innate immune responses. For example, the filoviral VP35 interferes with RLRs by either sequestering dsRNA as potential PAMPs or by binding to the RIG-I stabilizer protein activator of the interferon-induced protein kinase (PACT) to ultimately interfere with its activation [7]. Hantaviruses, on the other hand, remove the tri-phosphorylated termini of their genome to evade RIG-I recognition, highlighting a diverse role for viral enzymes to inhibit the activation of the IFN-induction pathway.

Besides directly interfering with the upstream stages of the IFN-induction pathway, hemorrhagic fever viral IFN-antagonists seem to target at least one other downstream step of this pathway, which often involves the prevention of IRF3 activation resulting in the lack of type I IFN induction. Similar to arenavirus NP, other HF viruses, such as filoviruses, hantaviruses or SFTSV (Severe Fever with Thrombocytopenia Syndrome Virus), also prevent the formation of the IRF3 phosphorylating TBK1-IKKε heterodimer.

Some of the HF-causing viruses can also interfere with the IFN-signaling pathway. It has been shown that Marburg virus VP40 inhibits the phosphorylation of STAT1, while Ebola virus VP24 blocks this pathway further downstream and prevents the nuclear translocation of the ISGF3 complex [7]. Dengue virus utilizes several of its proteins, NS2A, NS4A, NS4B and NS5, to antagonize IFN-signaling. The individual proteins downregulate the phosphorylation of Jak2 and STAT1, while targeting STAT2 for proteasomal degradation and thus, preventing any form of STAT-mediated signaling of this important antiviral pathway. It is important to note that inhibition of the IFN-signaling pathway has not yet been demonstrated during arenavirus infections.

The inhibition at multiple stages of the type I IFNs pathways by multiple proteins of several HF-causing viruses highlights the fact that these viruses utilize a multitude of strategies to achieve this. For many of human viruses, such as HF-causing viruses, the inhibition of early innate immune pathways is essential to optimize viral replication and transmission. A better level of understanding of the immunosuppression and immune evasion strategies by these lethal HF viruses may help develop the necessary preventative and treatment options.
